# Changing Emissions and Atmospheric Chemistry: Ongoing Impacts on Air Quality and Climate

**DOI:** 10.1021/acs.est.6c01723

**Published:** 2026-02-13

**Authors:** Chaoyang Xue, Jonathan Abbatt, Hendryk Czech, Kevin Jones, Imad El-Haddad

**Affiliations:** † Aerosol Chemistry Department, 28309 Max Planck Institute for Chemistry, Hahn-Meitner-Weg 1, 55128 Mainz, Germany; ‡ Department of Chemistry, University of Toronto, Toronto, Ontario M5S 3H6, Canada; § Institute of Chemistry, University of Rostock, Albert-Einstein-Strasse 27, 18059 Rostock, Germany; ∥ Lancaster Environment Centre, 4396 Lancaster University, Lancaster LA1 4YQ, U.K.; ⊥ PSI Center for Energy and Environmental Sciences, Paul Scherrer Institute, 5232 Villigen, Switzerland; # College of Environmental Sciences and Engineering, Peking University, Beijing 100871, China

**Keywords:** atmospheric chemistry, climate, air quality, emission control, reactive nitrogen, aerosol, POPs

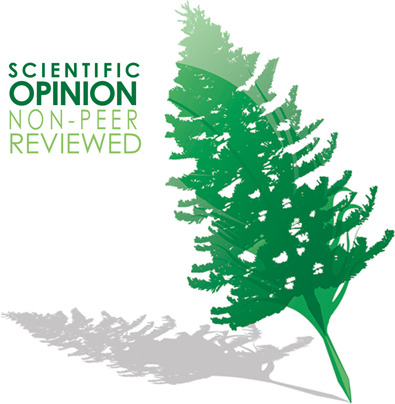

## Graphical abstract

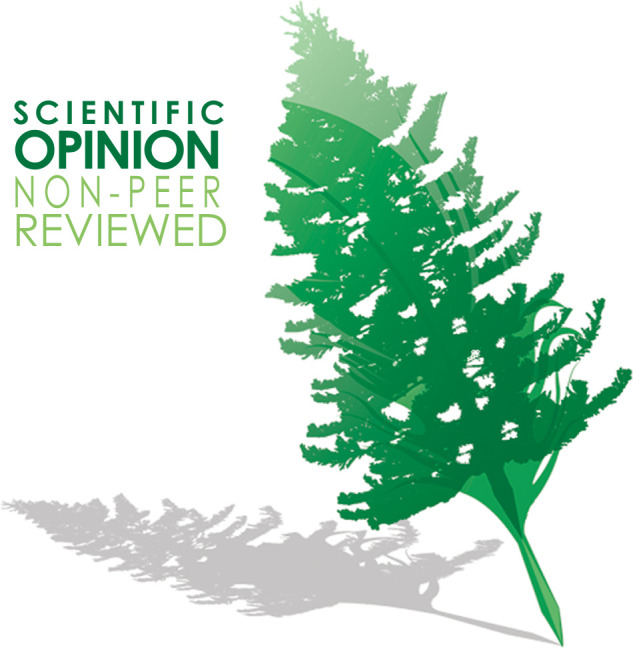

Human activity has profoundly altered atmospheric composition, leaving an unmistakable fingerprint on the Earth’s system, with investigations into atmospheric chemistry a core research topic at *ES&T* over many decades.^1^ An overarching theme in air quality research has been oxidation chemistry of primary emissions. For example, ozone formation occurs via oxidation of volatile organic carbon (VOC) species in the presence of nitrogen oxides (NO_*x*_). Secondary aerosol formation occurs through oxidation when sulfate forms from sulfur dioxide, nitrate from NO_*x*_, and highly oxidized organic molecules form from both biogenic and anthropogenic hydrocarbon emissions. Understanding this oxidation chemistry is the foundation for regulatory actions, which have led to remarkable improvements in air quality in many cities around the world. In the US alone, 2 trillion US dollars of economic value arise each year from improvements in air quality via the Clean Air Act.^2^ *ES&T* has been an important partner in this research area by supporting impactful and direction-changing research which ultimately has resulted in policy change and global environmental improvements.

However, major changes in atmospheric chemistry are currently underway. Greenhouse gas emissions continue to rise globally, and aerosols and their precursor vapors have declined in many regions as a result of air-quality regulation. Advances in exhaust after-treatment are dramatically reducing primary emissions of regulated species, particle mass, and black carbon. With the ongoing energy transition from fossil to renewable fuels, large classes of hydrocarbon emissions, such as aromatics and alkanes from gasoline and diesel, will decline in abundance, along with reductions of NO_*x*_. As a consequence, the relative importance of biosphere-atmosphere exchange of reactive nitrogen (e.g., soil-air exchange) in atmospheric chemistry is expected to increase.

In addition, commercial chemicals such as solvents, personal care products, pesticides, etc., will remain in the atmosphere, either with a petrochemical source or increasingly from sustainable feedstocks. Persistent organic pollutants (POPs) such as organochlorine pesticides, polychlorinated biphenyls (PCBs), dioxins, and more recently, per- and polyfluorinated alkyl substances (PFAS) will continue to undergo long-range atmospheric transport and redistribution around the globe. Also of consideration is that while aerosols and their precursor vapors have declined in many regions, this carries a climatic trade-off because aerosols have partially masked greenhouse-gas warming by scattering sunlight and modifying cloud properties. As aerosol levels fall, this cooling shield weakens, potentially accelerating warming. Understanding how concurrent changes in greenhouse gases, aerosols, and natural emissions reshape clouds, radiation balance, and atmospheric chemistry has therefore become a central challenge in climate science. This viewpoint examines the atmospheric chemistry transition currently underway with insights on potential unintended outcomes on air quality and climate, and future research needs.

## Emission Controls Are Reshaping Air Pollution Chemistry and Environmental Exposure

Advances in combustion engine exhaust after-treatment technologies have dramatically reduced primary emissions of regulated species, particle mass, and black carbon over the past few decades. This includes three-way catalysts, oxidation catalysts, diesel particulate filters, and gasoline particulate filters resulting in engines emitting far fewer primary particles. Filter after treatment has significantly lowered primary organic aerosol (POA) and elemental carbon mass.^3–5^

However, these after-treatment technologies do not necessarily eliminate secondary aerosol formation. Modern exhaust-compliant vehicles that meet primary particulate matter (PM) or particle number regulations can still emit a complex mixture of volatile and intermediate-volatility organic compounds (VOCs/IVOCs), NO_*x*_, ammonia, and other trace gases that once released into the atmosphere can undergo photochemical oxidation, nucleation and condensation. For example, smog chamber experiments revealed that diesel emissions with after-treatment produce substantial secondary organic aerosol (SOA), often many times the mass of any residual POA.^6–8^ Modern vehicles compliant with recent emission and after-treatment standards have also shown that diluted exhaust still yields substantial SOA following atmospheric oxidation.^6,9,10^

This demonstrates that regulatory compliance in terms of primary PM mass or particle-number per km does not guarantee absence of aerosol formation postemission. Given that SOA contributes to fine particulate matter exposure relevant for human health, and given that secondary aerosol may even dominate total carbonaceous PM under modern vehicle fleets, these findings have major implications for future air-quality and public-health assessments.^11–13^

Looking forward, as the energy transition expands with the transportation sector and nonroad machinery shifting toward low- or zero-carbon fuels (e.g., ammonia/diesel blends, pure NH_3_, H_2_, biofuels) combined with advanced after-treatment, it becomes vital to re-evaluate emissions not simply on the basis of regulated primary PM or NO_*x*_, but through a full source-to-atmosphere-to-exposure lens.^14^ Key research priorities include: (1) systematically characterizing the full speciation (gases, IVOCs, SVOCs, ammonia, nitrogen-containing organic compounds) of exhaust from alternative-fuel and hybrid combustion systems under real-world operating conditions; (2) conducting smog-chamber or oxidation flow reactor experiments with these exhausts + realistic atmospheric oxidants to quantify SOA formation potentials; (3) measuring health- and climate-relevant chemical and physical properties (size distribution, volatility, hygroscopicity, oxidative potential) of secondary aerosols; and (4) coupling aerosol measurements with toxicological and epidemiological exposure metrics to assess potential health effects of combustion exhaust including both fresh and aged emissions. Only by bridging after-treatment chemistry, atmospheric transformation, and exposure science will we be able to judge whether next-generation “clean combustion” technology reduces total risk to human health and climate, or simply is successful in reducing regulated pollutants.

## Increasing Importance of Reactive Nitrogen Exchange at the Soil-Air Interface

In an era of declining anthropogenic reactive nitrogen (Nr) emissions, driven by the above-mentioned emission controls and other mitigation measures, Nr exchange at the soil-air interface is becoming increasingly important in atmospheric chemistry, particularly in the context of a changing climate. Early research has studied soil ammonia (NH_3_) volatilization as a major nitrogen loss pathway^15,16^ and an important precursor for aerosol formation.^16–18^ Soil nitrous oxide (N_2_O) emissions have also been extensively studied because of their potent contribution to global warming and to the depletion of stratospheric ozone.^19–21^

However, scientific focus is now shifting toward soil-emitted NO_*x*_ and, more crucially, nitrous acid (HONO). These species are recognized as pivotal drivers of atmospheric oxidizing capacity and regional air quality, particularly through their roles in modulating atmospheric oxidizing capacity, which in turn contributes to the formation of ozone and secondary aerosol. A number of studies based on field flux measurements, process-based modeling, and satellite inversions have been conducted to improve the global soil NO_*x*_ emission inventory^22–24^ that could be implemented into chemical transport models to quantify the impact on air quality (e.g., O_3_ and PM) and public health.^25–27^ Nevertheless, uncertainties persist in these inventories due to the complexity of the soil microbial processes and the lack of high-resolution input data. In contrast, HONO exchange at the soil-air interface is relatively poorly studied. On the one hand, similar to soil NO_*x*_ or N_2_O, HONO can be formed through biogenic pathways.^28,29^ On the other hand, heterogeneous reactions on the soil-air interface, such as NO_2_ reduction and nitrate photolysis, serve as an important abiotic source of HONO.^30–32^ However, key kinetic parameters, including NO_2_ uptake coefficients on different real surfaces and nitrate photolysis frequencies, remain highly uncertain,^33,34^ complicating their integration into model simulations.^35,36^ Further studies, including developing techniques for field flux measurements and laboratory experiments, are still needed to apportion these HONO sources and advance our understanding of their atmospheric impact.^37^

The use of nitrogen fertilizer is known to significantly enhance soil Nr emissions; however, the emissions show large temporal and spatial variabilities. This limits our understanding of its atmospheric impact, particularly for NO_*x*_ and HONO, which are photochemically active and therefore, possess a relatively short lifetime. Given the massive amount of nitrogen input to agriculture systems (e.g., 112 Mt in 2023 globally, data source: Food and Agriculture Organization of the United Nations), quantifying the precise emission factors of these Nr species across both temporal and spatial dimensions is essential for refining air quality models.^24,38^

Mitigating strategies to reduce soil NO_*x*_ and HONO emissions have been preliminarily explored, for instance, by using nitrification inhibitors^29,39^ or optimizing fertilizer application method.^40^ However, the comprehensive impact of those strategies on other Nr species (NH_3_, N_2_O, NO_*x*_, etc.) and the potential trade-offs between them remain poorly understood. Those mitigation strategies and other emerging ones need to be comprehensively assessed by laboratory and field experiments, with a critical focus on balancing environmental benefits against crop yields. Moreover, a deeper understanding of canopy’s reduction impact on soil Nr emissions is essential to better quantify its global impact on air quality or climate.^23^ Furthermore, declining anthropogenic emissions may reduce nitrogen deposition, which in turn could alter Nr emissions from natural systems (e.g., forest, wetland, etc.) and requires further study.^41^

## Shifting Pathways of Aerosol Production in a Cleaner, Warmer Atmosphere

The success of air-quality regulations has profoundly altered the atmospheric aerosol system. Over recent decades, emissions of sulfur dioxide, nitrogen oxides, and primary particles have declined across much of Europe and North America, with similar trends now emerging in parts of East Asia.^42^ These reductions have delivered major public-health benefits but have also weakened the aerosol cooling shield that has historically masked a fraction of greenhouse-gas warming. As aerosol burdens decline, new particle formation (NPF) and secondary aerosol growth play an increasingly dominant role in determining particle number, size, and cloud interactions. Understanding how these processes respond to changing emission regimes is therefore central to predicting future air quality and climate feedback.

New particle formation is responsible for roughly half of global cloud condensation nuclei in the present-day atmosphere and likely contributed an even larger fraction in the preindustrial atmosphere, with its importance expected to increase again in a future world characterized by low primary aerosol emissions.^43^ Prior to the past decade, sulfuric acid–driven nucleation dominated both conceptual models and global simulations, yet laboratory rates spanned orders of magnitude with some studies suggesting that sulfuric acid alone could explain ambient NPF and others finding rates far too low to be atmospherically relevant. A decisive turning point came from ultraclean, high-precision laboratory measurements, showing that NPF and early growth are governed by a broader chemical space than previously assumed. Sulfuric acid alone cannot account for observed particle formation,^44^ requiring stabilization by bases and ions and, in many environments, substantial contributions from organic vapors,^45^ nitric acid^46^ and iodine oxoacids.^47^ This mechanistic clarity resolved long-standing discrepancies between experiments and observations^48^ and established a framework in which NPF rates and survival probabilities are tightly coupled to emission-dependent chemical regimes.

Ammonia and, more efficiently, amines were identified as key stabilizing agents for embryonic clusters, explaining the strong sensitivity of NPF to agricultural and urban emissions.^49^ While historically associated with these sources, amines may become increasingly influential in a low-sulfur future, particularly if amine-based technologies are deployed at scale for carbon capture. Even modest increases in amine availability could substantially enhance sulfuric-acid-driven nucleation under otherwise sulfur-limited conditions, highlighting the tight coupling between emerging climate mitigation strategies and particle formation pathways.

An equally transformative discovery was the recognition that pure organic vapors can drive both particle formation and growth under low-sulfur conditions,^50–52^ providing a long-sought explanation for frequent NPF in remote continental regions.^53–56^ Upon the oxidation of biogenic emissions, rapid autoxidation of peroxy radicals^57^ and their accretion into dimers produce ultralow-volatility organic compounds capable of nucleating and sustaining early growth without inorganic acids. This mechanism implies substantially higher natural aerosol levels in the preindustrial atmosphere, reducing inferred anthropogenic aerosol radiative forcing. Looking forward, it also suggests that in a future atmosphere, characterized by low sulfuric acid but enhanced biogenic emissions driven by warmer temperatures and longer growing seasons, organic-driven NPF and growth may become increasingly important across large regions of the globe.

A parallel paradigm shift occurred in marine and polar regions with the recognition of iodine-driven NPF.^47,58^ Coastal and high-latitude observations repeatedly linked intense particle formation to iodine emissions,^59^ particularly in environments influenced by sea ice and macroalgae. Laboratory studies showed that iodine oxoacids nucleate extremely efficiently and can drive early growth through condensation at the kinetic limit. Unlike the sulfuric-acid–ammonia system, a single precursor vapor can sustain the full NPF-to-growth sequence, producing particles that rapidly reach climatically relevant sizes. Importantly, iodine emissions from the ocean surface are estimated to have tripled since the preindustrial era and are projected to rise further as ozone levels increase and sea ice retreats.^60^ Because cloud radiative forcing in remote marine and polar regions is strongly limited by CCN availability, even modest changes in iodine-driven NPF may exert disproportionate climatic impacts. Yet, iodine chemistry remains highly uncertain with possibilities of recycling from the particle phase back to the gas-phase sustaining NPF.^61^ Iodine emissions and chemistry are sparsely represented in global models,^62^ and long-term observational constraints over the open ocean are still limited. Understanding the trajectory of iodine emissions and chemistry in a changing environment represents a critical research priority.

Beyond nucleation, the growth of newly formed particles to CCN sizes often constitutes the true kinetic bottleneck controlling climatic relevance. A pressing challenge lies in understanding how secondary aerosol growth and particle survival will evolve in a future atmosphere characterized by lower sulfur and nitrogen oxides but higher ammonia, organic vapors, and temperatures. Declining sulfur and nitrogen oxide emissions fundamentally alter both gas-phase oxidation and multiphase processes, while shifting the balance toward organic-driven growth.

Central to this transition is the changing fate of organic peroxy radicals. As NO_*x*_ emissions decline, RO_2_ chemistry shifts away from reactions with NO toward reactions with HO_2_ and RO_2_, increasing RO_2_ lifetimes and enhancing autoxidation.^63,64^ This favors the formation of more highly oxygenated, lower-volatility products that can contribute efficiently to particle growth. At the same time, declining NO_*x*_ reduces global oxidant production,^65^ increasing sensitivity to natural NO_*x*_ sources such as lightning and biosphere-atmosphere exchange, which is expected to intensify in a warmer climate, and to aviation emissions in the upper troposphere. These changes are likely to have particularly strong consequences for particle formation aloft, where small perturbations in oxidant levels can strongly influence nucleation rates. These competing effects introduce strong nonlinearities in NPF and growth that will need to be better understood in a changing environment.

Declining sulfate and nitrate concentrations reduce aerosol acidity and lower aerosol water content, altering multiphase chemistry and growth through reactive uptake. For example, uptake of isoprene epoxydiol onto acidic sulfate may weaken, while nitrogen-containing organic pathways, including imidazole formation from small carbonyls in ammonia-rich environments, may be enhanced. These shifts imply that future aerosol growth will not simply scale with precursor availability but will reflect a qualitatively different chemical regime, as emissions evolve.

Observations from rapidly transitioning regions such as China illustrate the complexity of these interactions. Compared with gas-phase photochemical oxidation, which tends to be self-buffered under heavily polluted conditions, multiphase chemistry exhibits strong positive feedbacks, where elevated particulate matter enhances heterogeneous and aqueous-phase reactivity, further accelerating secondary aerosol production. In China, high concentrations of anthropogenic sulfate and nitrate, together with persistently high relative humidity, have historically created a particularly efficient medium for aerosol growth during haze episodes.^66^ Emission controls have reduced sulfate and nitrate burdens, most likely suppressing some heterogeneous pathways, while nonlinear ozone chemistry has increased oxidant levels in urban areas enhancing aerosol growth through condensation, including frequent NPF events observed at the onset of haze episodes.^67^ Similar trajectories are likely to occur in other highly polluted regions in the next few decades, including parts of South Asia and Africa, where NO_*x*_ mitigation may initially increase ozone and accelerate secondary aerosol production before longer-term declines set in. Understanding how NPF and growth respond along this transition pathway is essential for anticipating future aerosol burdens.

Superimposed on these trends is the growing importance of emerging emission sources. Volatile chemical products, originating from solvents, coatings, personal care products, and indoor emissions, have become major contributors to urban organic vapor budgets in regions with strict vehicle regulations.^68^ These emissions exhibit high and variable SOA yields and may contribute disproportionately to particle growth in urban regions with low combustion emissions. In parallel, biomass burning, often framed as carbon-neutral, emits complex organic mixtures that undergo rapid intraparticle light-induced reactions^69^ or form secondary aerosol through both daytime and nighttime oxidation.^70^ These multiple pathways complicate the attribution of biomass-derived particles in aged air masses, and their relative contribution to aerosol growth and composition remains highly uncertain.

As anthropogenic emissions decline, natural sources – biogenic volatile organic compounds, biogenic NO_*x*_ emissions, marine halogens, wildfires, and dust – will increasingly shape aerosol populations.^42^ Climate-driven increases in biogenic emissions with temperature and CO_2_ fertilization are expected to amplify organic-driven NPF and growth, although the net outcome will depend on competing changes in oxidant production and lifetime driven by anthropogenic emissions. Wildfires are clearly increasing in frequency and intensity, contributing episodic but substantial sources of condensable vapors and particles. The traditional dichotomy between “natural” and “anthropogenic” aerosols is therefore becoming increasingly blurred, as human activities alter the natural ecosystem itself and the chemical environment in which natural emissions are processed.

Beyond surface processes, the scavenging and transformation of gases and particles in clouds exert a powerful yet poorly constrained control on aerosol formation and growth, particularly in the upper troposphere. Cloud droplets and ice crystals regulate the vertical redistribution of key precursors such as ammonia and oxygenated organic vapors, modify their chemical lifetimes, and can either suppress or promote new particle formation depending on the balance between scavenging and in-cloud production.^71^ Despite their importance, these processes have remained difficult to quantify owing to the lack of experimental systems capable of reproducing realistic cloud formation and processing.

While near-surface oxidation of biogenic precursors produces some low-volatility products, most first-generation compounds remain sufficiently volatile to persist in the gas phase. Deep convection over tropical forests, boreal regions, and marine environments efficiently transports these oxidized vapors to the upper troposphere, where lower temperatures, continued oxidation, and cloud processing strongly reshape their fate. Recent laboratory^72^ and aircraft observations^73^ have demonstrated that oxidation products of even small, volatile molecules such as isoprene can nucleate efficiently at altitude—a finding of global significance given that upper-tropospheric NPF contributes up to ∼ 30% of global CCN and strongly influences cloud radiative properties in remote regions such as the Southern Ocean.

However, a central uncertainty lies in how cloud processing modifies these ascending vapors. New laboratory measurements have shown that many isoprene oxidation products are moderately to highly water-soluble and are efficiently scavenged during cloud formation, where aqueous-phase reactions substantially alter their volatility and reactivity, producing secondary organic aerosol yields far exceeding those of gas-phase pathways.^74^ When implemented in global models, this enhanced in-cloud production markedly increases biogenic SOA burdens, identifying cloud processing as a potentially major yet still underrepresented contributor to aerosol growth.

Despite this progress, the explored chemical regimes remain narrow. Current laboratory systems struggle to access subfreezing temperatures. Advancing this frontier will require new experimental platforms capable of generating subcooled droplets and ice crystals under realistic upper-tropospheric conditions, coupled with multiscale modeling approaches that link process-level parametrizations to convective transport and global climate impacts. As emissions continue to evolve, constraining vapor–cloud interactions will be essential for understanding particle formation and growth aloft and their implications for aerosol–cloud–climate feedbacks in a future climate.

Looking forward, the central challenge is no longer identifying individual particle production mechanisms, but predicting how NPF, secondary growth and aqueous processing reorganize under simultaneous changes in emissions, climate, and atmospheric composition. Key research directions include: constraining iodine emissions, recycling, and particle-phase chemistry in marine and polar environments; quantifying natural particle formation and growth in a warmer, chemically cleaner atmosphere; understanding aerosol formation and growth from emerging pollutants such as volatile chemical products and biomass burning; resolving the role of multiphase reactive uptake under evolving aerosol acidity and water content; and understanding the influence of hydrometeors on precursor transport, aerosol burden and composition. Addressing these challenges will require tightly integrated laboratory studies, long-term observations across emerging chemical regimes, and models capable of representing evolving particle production pathways. As emissions continue to change, understanding how particles are produced will be vitally important for predicting future air quality and climate.

## Persistent Organic Pollutants (POPs) in the Atmosphere: Decades of Change and Ongoing Challenges

While the success of air-quality regulations has led to the decline of certain atmospheric chemical species such as NO_*x*_ and PM, Persistent Organic Pollutants (POPs) by their very persistent nature are here to stay. POPs first started to attract scientific attention from the environmental science community in the late 1960s through their long-term environmental persistence and their occurrence and accumulation in the food chain. Early studies detected POPs in top predator marine and terrestrial biota (e.g., marine mammals, birds of prey) as well as humans in locations far from sources, such as the Arctic. This led to investigations of long-range atmospheric transport (LRAT) and associated atmospheric chemistry, gas-particle partitioning and air-surface interactions (e.g., Bidleman^75^ and Eisenreich et al.^76^). From the outset, *ES&T* was a major forum for much of this research.

Motivated by concerns for top predator species and potential human exposure, early ‘classic’ studies investigated atmospherically driven POPs cycling in the Great Lakes systems of North America, studying the air–water exchange processes and mass balances for the lakes.^77,78^ The discovery that POPs undergo repeated temperature-dependent air-surface exchange led to the fascinating global redistribution hypotheses involving ‘cold condensation’, ‘global fractionation’ and ‘grass-hopping’ mechanisms that resulted in POPs undergoing long-range transport to accumulate in colder remote parts of the planet.^79,80^ This spawned many *ES&T* papers that investigated and tested these hypotheses – exploring the role of primary and secondary emission sources of POPs to the atmosphere, the scavenging and retention of POPs from the air by forest systems (the ‘forest filter effect’), global sinks/retention in soil and sediment carbon stocks,^81–83^ and the ways in which atmospheric sources/processes can ultimately drive the link to food chain transfers and exposure for humans and wildlife.

The regulation and banning of POPs under international conventions occurred through the 1990s and 2000s. Country-level monitoring stations were established to assess the effectiveness of these regulatory-driven source reduction measures. In addition, scientific researchers in the Great Lakes, the Arctic and Europe established a limited number of atmospheric monitoring stations in the late 1980s/early 1990s. These monitoring programs have provided invaluable long-term data sets to define the sources of POPs, source reduction measures, and the processes controlling long-term trends (e.g., Venier and Hites^84^).

An early challenge for POPs researchers was to capture monitoring data in remote places, particularly in relatively pristine areas where trace air concentrations could be in the low pg – ng m^–3^ range. *ES&T* published many of the early papers on the development of passive air samplers (PAS), which enabled integrated long-term ambient concentrations to be obtained over many months and years.^85^ Regional and global background data were collected at many sites across the world and passive air sampling became an accepted way to collect data under the Global Monitoring Programme of the Stockholm Convention on POPs.^86^ These data sets are now often used in regional and global scale multimedia modeling studies, to explore and refine the links between source inventories, ambient measurements, environmental processes and chemical fates.^87^

Voluntary bans by industry, national and international control measures under the Stockholm Convention have generally led to reductions in atmospheric concentrations of the ‘legacy’ or traditional POPs – such as organochlorine pesticides, polychlorinated biphenyls (PCBs) and dioxins. Generally, there are ongoing uncertainties for emissions inventories of these legacy POPs, arising from diffusive primary sources, stocks of chemicals still in products, wastes or in ‘environmental reservoirs’, such as soils or sediments. However, POPs and POP-like compounds continue to be developed commercially with many new classes of industrial chemicals exhibiting POPs behavior of long-term environmental persistence. Perfluorinated compounds are perhaps the most important current example, because of their extreme persistence, the extensive range of their properties and reaction pathways, and the need to unravel the role of LRAT and global oceans/currents to understand their global-scale cycling.^88^ Other emerging POPs classes have also been added to the Stockholm Convention more recently, such as the chlorinated hydrocarbons and paraffins, while others are in active discussion for inclusion, as is the process for the addition of new substances.^86,89^ *ES&T* remains at the forefront in publishing these important advances in the field, as well as the wider context around POPs including internationally leading papers on improved prioritization, screening and management of organic chemicals, to protect the global system long-term.^88,90,91^

## Concluding Remarks

The dynamic nature of atmospheric chemistry and the constantly evolving chemistry of anthropogenic emissions will continue to demand advances in spatial and temporal measurement techniques and mechanistic understanding of reaction drivers leading to advances in more representative modeling. As illustrated in this article, while the advances in the combustion engine exhaust after-treatment technologies have successfully led to declining primary emissions, the oxidation of exhaust components continues to yield substantial secondary pollutants. Assessing the efficiency of future combustion technologies requires a shift from simple tailpipe compliance to a ‘source-to-exposure’ perspective. Integrating field and laboratory measurements of both fresh and aged emissions will be essential to accurately quantify the environmental and public health impact from the next generation of combustion technologies aiming to further reduce emissions.

Changing emissions will drive the increasing importance of soil-air and other biosphere-atmosphere exchange processes in atmospheric chemistry. Beyond well-documented roles of NH_3_ and N_2_O, soil-emitted NO_*x*_ and HONO are emerging as critical yet highly uncertain factors. As the overall NO_*x*_ level is going down, the atmospheric chemistry, particularly the O_3_ production, is becoming more sensitive to the abundance of Nr. However, the complex spatial and temporal emission characteristics of soil Nr emissions and their feedback to both nitrogen fertilizer and natural nitrogen deposition are still poorly constrained. As developing mitigating strategies for agricultural Nr emissions, comprehensive assessments are vital to understand the trade-offs between different Nr species and to balance the reduction goals with nitrogen use efficiency or crop productivity.

Changing emissions are also significantly shifting the drivers of atmospheric aerosol production, with NPF and secondary growth dominantly affecting aerosol properties and climate interactions. The mechanistic discovery that NPF is not governed by sulfuric acid alone but by diverse chemical species, including stabilizing agents such as ammonia, amines, nitric acid, and iodine oxoacids, along with the discovery of pure organic vapor-driven NPF and growth, has resolved long-standing discrepancies between laboratory investigations and field observations and helps explain the high natural aerosol levels in pristine and preindustrial atmosphere. Moreover, while the recognition of iodine-driven NPF advances the understanding of aerosol formation in marine and polar regions, open questions about iodine chemistry and its emission trajectory still lie ahead, limiting its integration into climate models. Furthermore, under future low-sulfur conditions, NPF will become more sensitive to biogenic and emerging anthropogenic emissions. However, the process remains highly nonlinear; the shift in peroxy radical (RO_2_) chemistry under low NO_*x*_ conditions, the transition toward naturally emitted organic-driven growth, and the complex interplay within cloud processing and multiphase chemistry create a multifaceted response to a cleaner, warmer atmosphere. Future research needs to prioritize these coupled chemical-physical feedbacks to accurately project aerosol-cloud-climate interactions in an era of evolving global emissions.

Unlike short-lived species such as reactive nitrogen and aerosols, which respond relatively quickly to emission controls, POPs exhibit a much slower atmospheric recovery. Given their environmental persistence and the mechanisms of global long-range transport, declining primary emissions are unlikely to result in a rapid reduction of POPs. Consequently, international conventions and voluntary industry bans are playing key roles regarding long-term management of POPs emissions and the reduction of their associated health impact. A similar proactive approach is essential for emerging POPs or POP-like compounds to avoid their widespread accumulation. Moreover, long-term monitoring of these species is critical to evaluate the efficiency of the regulation policies and their environmental impacts.

Indeed, there will be significant impacts across a wide range of fields, from urban air quality, to biosphere-atmosphere interactions, to clouds and climate, and to persistent organic pollutants. Important long-term goals are to map out the atmospheric chemistry and environmental and health impacts of all chemical species, given their large number but frequent low abundance, and to better decipher chemistry-climate couplings.
